# “It’s a feeling of complete disconnection”: experiences of existential loneliness from youth to older adulthood

**DOI:** 10.1186/s40359-023-01452-4

**Published:** 2023-11-21

**Authors:** Phoebe E. McKenna-Plumley, Rhiannon N. Turner, Keming Yang, Jenny M. Groarke

**Affiliations:** 1 Centre for Improving Health-Related Quality of Life, School of Psychology, Queen’s University Belfast, Belfast, UK; 2 Centre for Identity and Intergroup Relations, School of Psychology, Queen’s University Belfast, Belfast, UK; 3 Department of Sociology, Durham University, Durham, UK; 4 School of Psychology, University of Galway, Galway, Ireland

**Keywords:** Existential loneliness, Loneliness, Qualitative research, Life course Perspective, Emotions

## Abstract

**Background:**

Existential loneliness is a feeling which stems from a sense of fundamental separation from others and the world. Although commonly mentioned in the loneliness literature, there is relatively little empirical work on this construct, and existing work tends to focus on older and seriously ill individuals. The present study aimed to understand how people experience existential loneliness without specific constraints on precipitating factors like illness or age.

**Methods:**

A qualitative online survey collected data from 225 adults aged 16 to 72 years old. Participants were asked to write about their experiences of existential loneliness and how these experiences compared to non-existential loneliness. Data were analysed using reflexive thematic analysis.

**Results:**

Of 225 participants, 51% knew the meaning of “existential loneliness” upon accessing the survey and in total, 83% had experienced existential loneliness. 93% of these participants had also experienced loneliness that was not existential in nature. 175 participants provided qualitative data regarding their experiences of existential loneliness, from which four themes were identified: Existential loneliness is (1) A deeper form of loneliness, and (2) A feeling of deep disconnection, in which (3) Cognitive evaluations and negative emotions are central elements, and (4) Stress and mental health issues are perceived as relevant factors.

**Conclusions:**

Existential loneliness is a deeply rooted and impactful form of loneliness which involves feelings of profound separateness. This aspect of loneliness is deserving of further attention. Future research directions are suggested.

## Background

Loneliness is a distressing and painful experience which negatively impacts physical health, mental health, and wellbeing [1–3]. Although loneliness can occur when a person is socially isolated, it is a subjective feeling that one’s connections are lacking in some way which is separable from objective isolation [4]. Previous research indicates that loneliness may be more appropriately conceptualised as multidimensional, despite the dominance of a unidimensional approach in the loneliness literature [5, 6]. For example, a recent systematic review of qualitative studies on loneliness found that loneliness can be felt in a pervasive sense, but can also relate to deficiencies in specific relationships or relationship types [7]. This aligns with Weiss’s [8] social needs theory which proposes two dimensions of loneliness related to specific relational deficits: (i) social loneliness, which arises when one feels that they lack a sufficient social network, and (ii) emotional loneliness, which results from the lack of satisfying intimate relationships. Another dimension is existential loneliness, which describes a deeply rooted form of loneliness stemming from a sense of fundamental separation from others and the world [9]. A wealth of literature describes loneliness as including social, emotional, and existential types [10–17] and two studies of older adults provide empirical support for this three-dimensional model [6, 18], but the existential dimension has been relatively neglected in loneliness research. Existential loneliness may represent the pervasive type of loneliness evidenced by recent qualitative evidence synthesis [7]. It has been noted that efforts to address loneliness need to move beyond a ‘one-size-fits-all’ approach [19] and it is likely that different dimensions require different approaches – for example, increasing general social contact may not be effective for someone who is emotionally lonely and lacks a close, intimate connection. Given the adverse effects of loneliness on health and wellbeing, as well as substantial public health and policy interest in alleviating this issue, a comprehensive understanding of specific dimensions like existential loneliness is vital to improve efforts to address loneliness.

Existential loneliness has been the focus of writing by scholars and practitioners from existential [20], humanistic [21], and theological traditions [22], but it has been relatively overlooked in empirical work. This may be partly due to the challenges of operationalising the construct. Existential loneliness has been defined variably in the literature. Examples presented in Table 1 demonstrate the diffuse aspects of these definitions, although each definition describes a sense of fundamental separation. The lack of conceptual clarity has been noted in systematic and scoping reviews of the construct [13, 23]. In addition to challenges defining the construct, different approaches to labelling it may explain the relative dearth of empirical work on existential loneliness. While existential loneliness is discussed across the health psychology, nursing, and gerontology literature and by humanistic and existential writers such as Clark Moustakas and Irvin D. Yalom, the term existential isolation is also used by writers such as Yalom and in a growing area of existential psychological literature. Existential isolation research defines the construct as “feeling as though one differs, either chronically or acutely, with respect to one’s subjective experience” [24, p. 56]. This quantitative research has found associations with being a man [25], having a minority identity [26], insecure attachment [27], and negative outcomes including depression and suicide ideation [28]. This work focuses on the degree of shared perspective a person experiences, which may not exhaustively account for feelings of existential loneliness, and does not explicitly tap into the negatively valent aspects of loneliness indicated by scholarly writing in existential, humanistic, and theological traditions [20–22] and the existing qualitative literature [29–32]. A conceptualisation of existential isolation as an objective state and existential loneliness as a subjective feeling has also been employed [13, 33]. Research conceptualising existential loneliness as a felt experience of loneliness that impacts interpersonal relationships may complement this literature.

**Table 1:** Existing definitions of existential loneliness

Source	Definition
Moustakas ([21], p. 24)	“Existential loneliness is an intrinsic and organic reality of human life in which there is both pain and triumphant creation emerging out of long periods of desolation. In existential loneliness man is fully aware of himself as an isolated and solitary individual while in loneliness anxiety man is separated from himself as a feeling and knowing person.”
Yalom ([20], p. 221)	“An existential loneliness which […] extends far beyond ordinary social loneliness; it is the loneliness of being separated not only from people but from the world, as one ordinarily experiences it, as well.”
Mayers et al. ([29], p. 1184)	“A third form of loneliness, existential loneliness, has been defined as a primary and inevitable condition of existence (Burton, 1961; Mijuskovic, 1977; Moustakis, 1961) for which no permanent remedy can be found. Proponents of this form of loneliness believe that since all humans are born into a world where perfect communication with others is impossible and only death is certain, a basic sense of loneliness emerges.”
Ettema et al. ([13], p. 142)	“An intolerable emptiness, sadness, and longing, that results from the awareness of one’s fundamental separateness as a human being”
Bolmsjö et al. ([9], p. 1315, 1322)	“A feeling of being fundamentally separated from others and the world” “EL can be understood as the immediate awareness of being fundamentally separated from other people and from the universe, primarily through experiencing oneself as mortal, or, and especially when in a crisis, experiencing not being met (communicated with) at a deep human (i.e., authentic) level, and typically therefore experiencing negative feelings, that is, emotions or moods, such as sadness, hopelessness, grief, meaninglessness or anguish.”
Larsson et al. ([34], p. 1624)	“A basic sense of loneliness that occurs when we, as human beings, face that we are separated and alone in the world despite having other people around”
van Tilburg ([6], p. e335)	“Existential loneliness stems from the realization that a human being is fundamentally alone, with the accompanying emptiness, sadness, and longing.”
Fried (2019) in Prohaska et al. ([35], p. 277)	“Existential loneliness describes feeling separate from other people and society – a void within, and being aware of one’s own mortality, e.g. a sense of longing that cannot be sated through any type of social interaction; despite solid relationships, the person still feels empty.”

Qualitative research on existential loneliness to date has typically focused on older [15, 32, 36–39] and chronically or seriously ill [33, 40] populations, although one recent study includes adolescents [41]. Research with these groups depicts existential loneliness as involving feelings of not belonging [15, 32, 36–39, 41], feeling distant from meaningful relationships [15, 32, 36–39, 41], lacking meaning in life [15, 37, 39], and having concerns about frailty, death, and the future [36, 37, 41]. A systematic review of the literature suggests that existential loneliness can be characterised as a condition (people are fundamentally separate), an experience (of a specific component of loneliness involving a total lack of relatedness), and a process (wherein the negative experience is transformed into a positive one) [13]. Additionally, a more recent review suggests that existential loneliness involves two characteristics: the perception of oneself as inherently separate, and the emotional aspects that come alongside that perception [9]. While cross-cultural research is lacking, one study expresses consistency in descriptions across Chinese and Swedish older adults [37]. This research underlines the importance of considering existential loneliness within the larger loneliness and social wellbeing literature, given that it is a distressing experience of interpersonal separation which may have negative consequences for wellbeing and health. This is particularly important given that existential loneliness has been described as a universal experience [42, 43] which is at least somewhat culturally invariant [37] and may emerge in adolescence due to increased awareness of oneself as a separate being [44]. Indeed, recent research indicates the presence of low levels of existential loneliness, which were associated with feelings of general isolation, in young adults in Greece [45]. However, existing research on existential loneliness is limited by the focus on a specific life period and circumstances. A thorough conceptualisation of existential loneliness requires consideration of the lived experiences of a range of people, given that loneliness is a personal and subjective experience. Given the need to move beyond generic loneliness interventions [46], this knowledge may facilitate the development of more effective strategies to address specific aspects of loneliness.

A comprehensive understanding of how existential loneliness is experienced across the lifespan is lacking due to the relative dearth of literature on this construct and the focus on older and seriously ill individuals in the studies which have been carried out. Various definitions of existential loneliness exist (see Table 1), but there is a lack of knowledge regarding how individuals define their own experiences. Given that loneliness is a subjective experience, it is important that we ground our understanding of existential loneliness in first-hand experiences. This study aimed to address these aspects of the literature by gathering information regarding experiences of existential loneliness in individuals at various stages of the lifespan without specific constraints on contextual factors like illness or age. Additionally, this study aimed to explore perceived similarities and differences between experiences of existential loneliness and loneliness that is not existential in nature. Two research questions were investigated:

1. How do people describe their experiences of existential loneliness?
2. How do experiences of existential loneliness differ from experiences of loneliness that is not specifically existential in nature?

## Methods

### Design

A qualitative online survey was used to collect data. While face-to-face interviews and focus groups have traditionally been used for qualitative data collection, technological advances afford the opportunity for innovative methods such as online qualitative surveys. Methodologists have argued that qualitative survey data are unique from interview data; they are usually very focused and dense with information, so smaller volumes can contain larger amounts of data [47, 48]. Moreover, this method can facilitate disclosure and reduce the social desirability that may be inherent in face-to-face collection methods [47]. Online surveys can engender an enhanced sense of anonymity which is welcomed by participants [49], particularly for a sensitive and stigmatised topic such as loneliness, and provide opportunities to participate for those who might not engage in face-to-face data collection methods [50]. Moreover, they can generate openness regarding study design, allowing participants to comment on the appropriateness of the questions or wording [47]. With the aim to recruit widely to understand experiences of a sensitive and personal topic, an online qualitative survey method was therefore chosen for the present study.

Ethical approval was granted by the Research Ethics Committee in the Faculty of Engineering and Physical Sciences at Queen’s University Belfast.

### Ontological and epistemological approach

Critical realism underpins the present study. This approach asserts that the context of the social world filters what we can learn about a potential reality. It is believed that participants’ descriptions of their experiences of existential loneliness are grounded in an experiential reality – they are accurate – but they are not independent from their context [51].

In terms of epistemology, the research is contextualist. This basis assumes that context is part of knowledge generation, such that all activity is situated within a sociocultural network of meanings [52]. Reality is an active, dynamic event rather than a single truth [53]. Knowledge is always incomplete but still grants us insight into the world.

### Recruitment

Participants were recruited via advertisements on social media (Twitter, Reddit), John Krantz’s ‘Psychological Research on the Net’ web portal, researchers’ networks, posters, and emails to relevant groups (e.g. university philosophy societies). These posts briefly explained the study and provided a link to the online survey. Participants had to be over the age of 16; they did not need to be aware of the meaning of the term “existential loneliness” at the outset of the study.

Decisions regarding sample size were guided by Braun and Clarke’s [54, p. 211] guidance that, having estimated an anticipated sample size range, “researchers should then make an in-situ decision about the final sample size, shaped by the adequacy (richness, complexity) of the data for addressing the research question”. We anticipated that up to 100 participants may be appropriate, but this was exceeded due to high interest from participants. PMP regularly checked the survey responses to assess whether a sufficiently rich and complex body of data had been collected; the survey was closed when the data appeared sufficiently rich and nuanced.

### Procedure

**Fig. 1:**
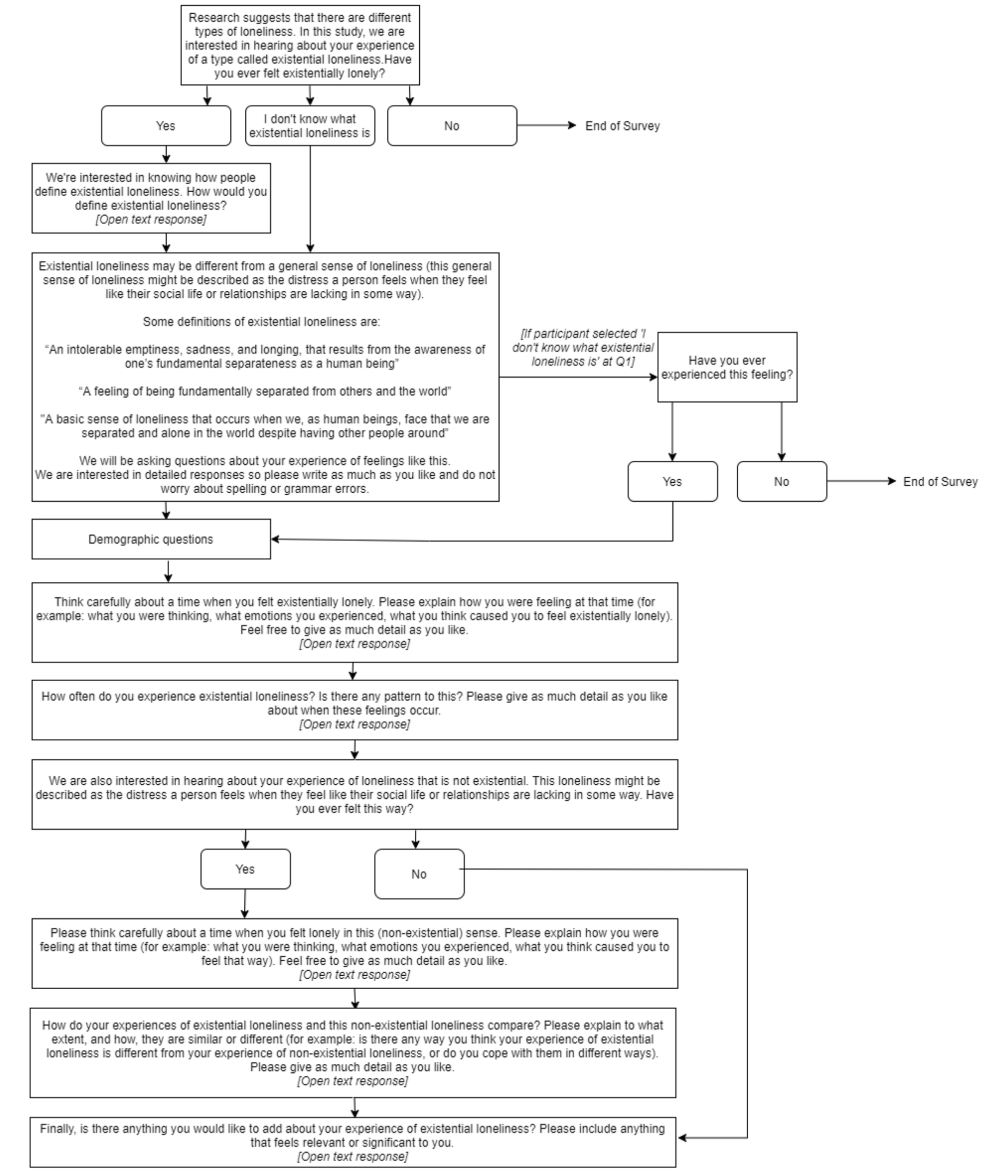
Questions presented to participants in the online survey

The online qualitative survey was piloted with three potential participants (1 woman, 2 men, aged 26–33 years old) and edited in line with their feedback on comprehensibility and comprehensiveness. The final survey questions are provided in Fig. 1.

Upon accessing the online survey, participants were presented with an information sheet, consent form, and six-digit code which could be used to later remove their response if they wished to withdraw. No participants requested to withdraw. Participants were then asked if they had experienced existential loneliness. If they stated yes, they were asked to provide their definition of existential loneliness. If they stated no, they were taken to the end of the survey. If they stated “I don’t know what existential loneliness is”, they were taken to a page which provided three definitions of existential loneliness from existing research [9, 13, 34; see Fig. 1]. They were then asked again if they had experienced this feeling, at which point they were taken to the end of the survey if they stated “no”. Participants who had stated yes originally were presented with these definitions after providing their own definition so that all participants had the same information. Following this, demographic questions were presented and then participants were asked to think carefully about a time they had felt existentially lonely and give open-ended responses to questions about this experience.

Participants were then asked if they had ever experienced loneliness that was not existential in nature, with an example provided of the distress one feels when they feel like their social life or relationships are lacking (see Fig. 1). If they stated yes, they were asked to think carefully about this experience and answer open-ended questions about the experience and how it compared to experiences of existential loneliness. Finally, all participants were asked to add anything relevant or significant about their experience of existential loneliness and thanked for participating. A list of resources for support organisations (e.g. the Samaritans) was provided.

### Analysis

The qualitative data analysis utilised reflexive thematic analysis, which is a flexible analytic approach in which themes are generated through iterative and reflexive engagement with the data [55]. This method was chosen as it allows the identification of overarching patterns in data regarding experiences [56] which is the aim of this study.

The analysis process followed guidance from Braun and Clarke [55, 57]. This involved repeated close reading of the data to become familiar with key points. Points of interest and early ideas were noted. The data were then thoroughly coded by PMP, a PhD candidate with advanced qualitative training and experience, with points which could be relevant for the research questions assigned one or multiple codes. Data were coded from the first participant onward and then a second time working backwards from the midpoint of the dataset to check, add to, and refine codes. Codes were checked to ensure their coherence, appropriate labelling, and thorough representation of the data without undue influence from the researcher’s preconceptions, for example that existential loneliness may stem from ideas about fundamental separation. Following this, the codes were organised into themes which explained important patterns in the data relevant to the research questions. Some themes also included subthemes, representing distinct but related aspects of the larger theme. Themes and subthemes were reviewed by PMP and JG to ensure their coherence, distinctiveness from other themes, and representation of the dataset. Finally, the full dataset was read again to check that the themes represented the data well.

## Results

### Participants

Data were collected from 225 participants. Of these, 186 stated that they had experienced existential loneliness and were therefore invited to complete the qualitative survey. Of these, ten participants answered none of the open-ended qualitative questions and one reported in the qualitative questions that they had never experienced existential loneliness, so these eleven participants were excluded from qualitative analysis. Therefore, 175 participants were included in the qualitative analysis. The qualitative responses ranged from 5 to 2,036 words total (*Mdn* = 142.5).

These 175 participants’ ages ranged from 16 to 72 years (*M* = 26.38, *SD* = 11.77); 129 were younger adults (16–29 years old), 40 were middle-aged adults (30–59 years old), and 6 were older adults (60 + years old). The majority identified as female (69.14%, *N* = 121), 44 (25.14%) identified as male, 10 (5.71%) identified another gender identity (including non-binary, genderfluid, and agender), and one preferred not to say. Most participants were located in the United States of America (*N =* 109, 62.29%), Northern Ireland (*N =* 20, 11.43%), England (*N =* 10, 5.71%), Canada (*N =* 10, 5.71%), and the Republic of Ireland (*N =* 7, 4%), while a smaller number were located in Germany, Australia, Wales, France, Guatemala, Israel, the Netherlands, Panama, the Philippines, and other parts of the United Kingdom. The majority (*N* = 108, 61.71%) identified themselves as White, while 19 (10.86%) identified themselves as Latino/Hispanic, 12 (6.86%) as Black, 11 (6.29%) as East Asian, and a smaller proportion as a mixed ethnicity, South Asian, Middle Eastern, another ethnicity, or preferred not to say. Most (*N* = 162, 92.57%) had completed secondary school or a higher level of education. The majority were employed (*N* = 103, 58.86%) and/or studying (*N* = 112, 64%), while 6 (3.43%) were retired and 6 (3.43%) were not employed or studying.

### Knowledge & prevalence of existential loneliness in the sample

Approximately half of the individuals who accessed the survey knew the meaning of the term “existential loneliness” (*N* = 115, 51.11%). Of those, 86.96% (*N* = 100) had experienced the feeling. 78% (*N* = 86) of those who did not initially know the meaning of the term “existential loneliness” also identified as having experienced it after being presented with definitions of the construct. In total, the majority of participants (*N* = 186, 82.67%) had experienced existential loneliness. Almost all individuals who had experienced existential loneliness had also experienced loneliness that was not existential in nature, but 13 (6.99%) had not.

### Definitions of existential loneliness

Definitions of existential loneliness were sought to check that the term “existential loneliness” was conceptualised in a congruent manner by participants and the research team. Ninety-three participants included in the qualitative analysis gave a definition. These were coded inductively to create a summary of major elements. Participants’ definitions described existential loneliness as a type of loneliness that revolved around feeling alone, disconnected and separate from the world and everyone around you, that was not related to isolation, was deeper and cosmic in scale, included a sense that one could not be understood by or fully share their experiences and thoughts with others, and involved a lack of meaning in life and disconnection from greater purpose. Some definitions indicated that existential isolation (our fundamental aloneness in the universe) was a fact or a thought, but existential loneliness was generally defined as a feeling. These definitions generally aligned closely with the definitions which are available in existing literature, although a lack of purpose was more prominently mentioned by participants in the current study. This indicates that lay conceptualisations of existential loneliness are similar to those used in the academic literature and that participants were writing about experiences of the same phenomenon that we aimed to explore. A comparison of major elements of existential loneliness related in definitions given in this study and in the extant literature is given in Table 2.

**Table 2:** Major elements of provided definitions and comparison to existing definitions

Major elements of definitions provided by participants	Excerpt from participants’ definitions	Excerpt from definitions in the literature (emphasis added)
Feeling fundamentally alone	“Feeling as though you are alone in this existence” (P35, 17-year-old, female) “Existential loneliness is the feeling of being alone within the universe that no amount of social interactions can change.” (P51, 21-year-old, female)	“[A]n everpresent feeling of aloneness experienced by human beings” ([33], p. 95)
Disconnection from the world and other people	“Feeling like you’re disconnected from the real world and everyone around you” (P22, 21-year-old, male) “[T]he feeling of loneliness that can never be fixed by people around you due to feelings of detachment” (P151, 18-year-old, genderfluid)	“A feeling of being fundamentally separated from others and the world” ([9], p. 1315)
Not related to social isolation	“[H]aving the company of others but still feeling separated from everybody else” (P133, 18-year-old, female) “Feeling like no one relates or understands to my internal thoughts, feelings, and worldviews - not just feeling that I can’t find friends or a partner to spend physical time with. I can feel ‘existentially lonely’ in a room full of people.” (P87, 37-year-old, female)	“A basic sense of loneliness that occurs when we, as human beings, face that we are separated and alone in the world despite having other people around” ([34], p. 1624)
A deep form of loneliness	“A deeper form of loneliness without an obvious cure” (P46, 36-year-old, female) “Loneliness in a broader scale, especially when it comes to a larger, particularly cosmic scale” (P38, 17-year-old, male)	“An existential loneliness which […] extends far beyond ordinary social loneliness” ([20], p. 221)
Inability to be understood and fully share thoughts and feelings	“Feeling like no one relates or understands to my internal thoughts, feelings, and worldviews” (P87, 37-year-old, female) “[T]hat feeling of knowing that you can never fully see exactly who anyone else is because you cant live in their own vivid individual world.” (P12, 20-year-old, female)	“[S]ince all humans are born into a world where perfect communication with others is impossible and only death is certain, a basic sense of loneliness emerges.” ([29], p. 1184)
Lack of meaning in life	“A lack of true meaning in life.” (P124, 19-year-old, female) “Feeling completely alone in the face of an empty and infinite universe, with nothing meaningful to which i can cling” (P54, 23-year-old, female)	“[A]nd typically therefore experiencing negative feelings, that is, emotions or moods, such as […] meaninglessness or anguish” ([9], p. 1322)
Lack of purpose	“I would define existential loneliness as one losing sight of their purpose in life” (P144, 23-year-old, male) “A feeling of not belonging because of a lack o[f] purpose” (P86, 59-year-old, male)	Purpose is generally not explicitly mentioned in definitions; however, it is included in measures [29] and other qualitative findings [32] on existential loneliness.
Existential isolation as a fact/thought	“[E]xistential loneliness is having the idea of ​​being alone in the world” (P139, 17-year-old, female) “[T]he feeling of fear and sadness I get when I think about how I exist in the universe alone” (P131, 38-year-old, female)	“Existential loneliness is an intrinsic and organic reality of human life” ([21], p. 24)
Existential loneliness as a feeling	“Feeling that you are alone and will eventually die alone” (P27, 18-year-old, female) “Feeling completely alone in the universe” (P28, 18-year-old, female)	“Existential loneliness describes feeling separate from other people and society” (Fried (2019) in Prohaska et al. ([35], p. 277)

### Qualitative analysis

In the process of characterising existential loneliness, it is relevant to note that some participants described it as difficult to define and mentioned that it could overlap with loneliness that was not existential in nature (*“In ways there are similarties [sp] as […] with both I feel a sense of isolation but also that sense of wanting to feel wanted and needed.”* – P55, 17-year-old, female), although most participants distinguished between forms of loneliness (*“For me, existential lonelieness [sp] is a far different can of worms compared to general loneliness.”* – P106, 17-year-old, male) and provided rich descriptions of their experiences.

**Fig. 2:**
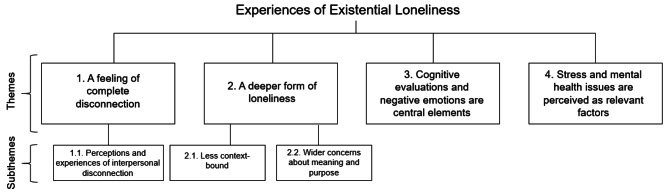
Visual representation of themes and subthemes

The process of reflexive thematic analysis ultimately produced four themes. Some include subthemes (indicated by subordinate numbering in Fig. 2, e.g. 1.1., 2.1., 2.2.), which express distinct but cohesive parts of the theme. These themes are described below and visualised in Fig. 2.

## A feeling of complete disconnection

In the participants’ accounts, experiences of existential loneliness involved feelings of complete disconnection.

*“[I]t’s a feeling of complete disconnection. I don’t know how to resolve it.”* (P46, 36-year-old, female).

This was described as feeling alone in the world, feeling an essential separation from other people, and in some cases as feeling disconnected from the world and from oneself. Existential loneliness appeared to constitute a feeling of essential aloneness and separation.

*“It made me feel separated entirely from society and other people. It was upsetting to realise that other people would not be able to relate to, or understand, my experiences and perceptions.”* (P70, 18-year-old, nonbinary).

Others described this more simply as feeling separated from the people around them. People experiencing existential loneliness often felt that others did not care about them, that they were alienated from other people, and that they did not belong.

*“I felt completely alone like there was no one there that would ever love me or care about me.”* (P155, 20-year-old, female).

These experiences were often linked to being different from other people and feeling that one was not understood by others. This could be due to specific aspects of identity, to certain experiences, or to personality factors. In a broader sense, people touched on the inability to fully know or be fully known by another person, and the impossibility of being fully understood.

*“I felt like no one else had lived my life, so no one else could relate to the experiences I had.”* (P63, 25-year-old, female).

For some people, feelings of disconnection were so intense that they provoked derealisation.

*“Felt like I was sort of outside of my body/the situation”* (P61, 30-year-old, female).

Several participants also noted a longing for a deeper relationship with another person.

*“[I]ts mostly a deeper longing for true soul connection that I experience, which I would equate more to existential loneliness.”* (P87, 37-year-old, female).

One subtheme was developed in connection with this theme:

### Perceptions and experiences of interpersonal disconnection

In describing experiences of existential loneliness, participants often described negative interpersonal experiences or perceptions. People described feeling inadequate or wrong.

*“All of this combined caused me to think why can I not be normal. I felt as though everyone was on some new kind of programming and I hadn’t received the update.”* (P74, 21-year-old, female).

There were also descriptions of being left out, not fitting in, and having no one to speak to. Participants described feeling invisible and unwanted, which may represent causes or symptoms of their feelings of disconnection.

*“I felt invisible and alone”* (P166, 60-year-old, female).

## A deeper form of loneliness

Existential loneliness was depicted by participants as a form of loneliness which was more pervasive, deeply rooted, and impactful. This also involved descriptions of existential loneliness as a deeply bad experience which was *“debilitating”* (P3, 48-year-old, female), *“far worse”* (P31, 17-year-old, male), and *“like a punch in the gut”* (P72, 18-year-old, male).

*“Existential loneliness feels like I’m alone in a void that only I can see and feel and it doesn’t exist or matter to others, however non-existential loneliness just makes me feel sad.”* (P151, 18-year-old, genderfluid).

This was described in terms of feelings and, for some, a statement of fundamental separation as a fact. Some participants characterised existential loneliness as universal or inevitable.

*“I think it was so overwhelming because it’s less a temporary state of being and more like an inherent part of human life. I think life is a distraction from the fact that we are all alone fundamentally (regardless of how depressing that sounds!).”* (P8, 24-year-old, female).

This deeper aspect of existential loneliness was also represented through descriptions of it as longer-lasting or in some cases permanent. Existential loneliness would persist, lasting days, weeks, or longer, as opposed to other forms of loneliness which were depicted as more fleeting. Indeed, some reported existential loneliness as a constant. Accordingly, experiences of existential loneliness often involved a sense of hopelessness or endlessness.

*“I felt completely lost and overwhelmed, like there was no light at the end of tunnel.”* (P53, 29-year-old, female).

Relatedly, participants described existential loneliness as difficult or impossible to resolve. It was harder to deal with than non-existential forms of loneliness, which could be eased through social connection. However, a small number of participants reported ways of managing existential loneliness through religion, positive thinking, gratitude, and shared experiences with others. When this was possible, positive outcomes such as acceptance and a better understanding of oneself were described by some participants.

*“Looking For ANYTHING to be thankful for even it were as simple as air to breath [sp] or the ability to blink, see and hear…… That is how I escaped being in that position.”* (P93, 53-year-old, female).

Additionally, some participants indicated that existential loneliness coincided with experiences of loneliness that was not existential and suggested that other forms of loneliness may lead to existential loneliness when they become chronic, further indicating the characterisation of existential loneliness as a deeper form of loneliness.

*“I think it would be easy for non-existential loneliness to culminate over a long time into existential, which has happened to me before.”* (P116, 21-year-old, female).

Two subthemes were developed as part of this theme:

### Less context-bound

Alongside descriptions of existential loneliness as longer-lasting was the experience of existential loneliness as less context dependent than other forms of loneliness.

*“The existential loneliness I feel seems to always be inside me, but it can come to the surface when I go too long to try to remedy it. Non-existential loneliness is situational and usually tied to a certain date or event.”* (P42, 32-year-old, female).

Existential loneliness was often depicted as lacking a clear cause – it was internal or constant, rather than arising due to a specific situation.

*“It happens randomly. I will be happy one moment and then lonely the next.”* (P85, 16-year-old, female).

However, while participants described existential loneliness as less context-bound, several interpersonal contextual elements did seem to drive experiences of existential loneliness: experiencing objective isolation, lacking social support, and periods of aloneness and, conversely, periods of socialising. Nonetheless, participants stressed that existential loneliness could arise despite strong social connections.

*“Existential loneliness is like sitting in your room with a phone full of messages and phone calls from loved ones still feeling like you are completely alone in the world”* (P159, 21-year-old, female).

Events which precipitated mental distress were also mentioned as relevant contextual factors for existential loneliness; see theme 4 for a full discussion.

### Wider concerns about meaning and purpose

Existential loneliness was described as an experience in which many people reflected on meaning and purpose in life, particularly concerns about lacking meaning or purpose. This differed from other forms of loneliness, which were generally described more straightforwardly with respect to social and interpersonal relationships.

*“Existential loneliness stems from me feeling like I’m going through the motions of life like a robot and my life is meaningless without connection to ground me”* (P171, 29-year-old, male).

In this respect, existential loneliness appeared to involve or emerge in light of wider existential concerns about one’s place and purpose in the world. Indeed, some participants remarked that finding or reaffirming their purpose in life eased feelings of existential loneliness.

*“Finding religion, love (romantically and socially), and an overall sense of purpose are essential for decreasing the power and frequency that existential loneliness can have on someone.”* (P158, 22-year-old, male).

## Cognitive evaluations and negative emotions are central elements

As described by the participants, existential loneliness appeared to involve cognitive and emotional facets: it was commonly linked to thinking or reflecting and was regularly described as involving sadness and other negative emotions. The experience was often described in times of thought or reflection. Several participants also described existential loneliness in respect to “overthinking”.

*“I[t] typically comes about when I’m having a bad day or when I just start thinking a bit too much about life.”* (P106, 17-year-old, male).

In some cases, these thoughts lingered on purpose in life, death, and the inevitability of being alone in the end. People often described feelings of insignificance alongside existential loneliness. Social comparison was another cognitive evaluation that was linked to existential loneliness, with descriptions of thoughts about being less connected or socially active than others.

*“I imagined every room having people my age enjoying warmth and company. I could see myself finally arriving at my hall and knowing no one from the foyer to my room.”* (P47, 72-year-old, male).

Negative emotions also featured in descriptions of existential loneliness. Existential loneliness was described as involving sadness and in some cases fear, dread, anger, and frustration.

*“It was just sadness and frustration feeling alone, in a crowd of people.”* (P85, 16-year-old, female).

Emptiness also characterised experiences of existential loneliness.

*“I just remember the fear of wondering will I always feel this empty inside and if one would be there for me or with me.”* (P99, 19-year-old, female).

### Stress and mental health issues are perceived as relevant factors

Mental health issues, stress, and trauma appeared to be relevant to the experience of existential loneliness for participants. Participants regularly described feeling existentially lonely during times of mental ill health and mentioned mental health issues in discussing their experiences.

*“I thought it was something more specifically triggered by my anxiety or depression.”* (P131, 38-year-old, female).

Stress was also described as a driver of existential loneliness. Participants felt existentially lonely in times of stress, after difficult days or life periods, and following life transitions which may constitute stressful events.

*“I have noticed it occurs when I go through some sort of prolonged stress in my life.”* (P53, 29-year-old, female).

Additionally, traumatic life events and experiences of abuse were described as pertinent to existential loneliness. These included abusive relationships, assault, childhood neglect and maltreatment, and unspecified traumatic experiences.

*“They can be triggered by periods of my life when I’m struggling with trauma of past experiences”* (P60, 19-year-old, male).

In some cases, periods of generally strong emotion were described as precipitating existential loneliness. This may be linked to descriptions by a small number of participants that existential loneliness was a feeling that was specific to their adolescence or early adulthood.

*“Now I look back on my young self and feel sad for her, but would like to tell her that it will be all right!”* (P165, 64-year-old, female).

## Discussion

This is the first research, to our knowledge, which specifically explores lived experiences of existential loneliness without constraints on sample characteristics like age group or health. Experiences of existential loneliness in people from 16 to 72 years old were investigated via an online qualitative survey. Additionally, this research sheds light on knowledge, prevalence, and definitions of existential loneliness using data from 225 adults. While these individuals were recruited to a study on existential loneliness and may therefore have a higher likelihood of knowing the term, just over half (51%) initially knew what existential loneliness was and 83% of all participants had experienced the feeling. The majority of these individuals had also experienced other forms of loneliness but 7% had experienced only existential loneliness. Data from 175 individuals with lived experiences of existential loneliness characterised it as (1) a deeper form of loneliness, and (2) a feeling of deep disconnection, in which (3) cognitive evaluations and negative emotions are central elements, and (4) stress and mental health issues are perceived as relevant factors.

### Defining the experience of existential loneliness

This study aimed to ascertain how people describe their experiences of existential loneliness and found that it was described as a deeper feeling of loneliness involving a sense of profound disconnection from other people, as well as in some cases from the world and from oneself. This study strengthens the conceptualisation of existential loneliness by adding empirical evidence of how individuals define and describe the construct. This is important given the lack of conceptual clarity regarding existential loneliness [13] and the relatively small amount of research on this dimension compared to social and emotional loneliness. It appeared that the central feature of existential loneliness experiences was a sense of profound disconnection from others; this is echoed in existing literature which characterises existential loneliness as “a total lack of relatedness” [13, p. 157] and “a feeling of fundamental separateness from others and the wider world” [14, p. 36]. While existing literature is inconsistent with regard to the definition and boundaries of this construct, through the present research, we suggest a core definition of existential loneliness as *a negatively valent feeling of profound aloneness and separation from other people*. Attempts to encapsulate the meaning of a construct may inevitably objectify and simplify it [58], but we suggest that this bottom-up conceptualisation, which centres lived experiences identified as existential loneliness, may be useful for research and practice. The present findings underline the complex, multidimensional nature of loneliness and indicate that attempts to manage loneliness will benefit from addressing feelings of deeper disconnection rather than solely providing opportunities for social interaction.

Existential loneliness was described as worse or more debilitating than other loneliness experiences, indicating the importance of considering this subgroup of experiences and their potential impact on wellbeing. Indeed, these experiences were described as involving negative emotional and cognitive characteristics such as sadness, dread, emptiness, and overthinking. This expands on previous research with older adults which indicates feelings of fear, guilt, and questioning life choices [36, 38, 39]. This research also noted a lack of purpose, which may link to behavioural outcomes if lacking purpose leads to a lack of motivation or action to pursue goals, interests, or responsibilities. Cognitive appraisals of social relationships are also a key facet of loneliness theory [59] and maladaptive social cognitive processes such as social hypervigilance appear to arise in response to loneliness [60], but this study suggests that reflecting or overthinking are also perceived as relevant for existential loneliness. The centrality of reflection and overthinking also indicates a potential role of rumination, which involves prolonged repetitive negative thinking and is associated with hopelessness, depression, and suicidality [61]. Existential loneliness may be related to a person’s awareness or belief in their fundamental separation from others, which may account for the heavier weighting of thought processes as a precipitating factor and the perception of it as a deeper experience.

This depth was also related to the perception of existential loneliness as longer-lasting, harder to resolve, and in some cases constant. This is concerning given that longer-lasting or recurring experiences of loneliness are associated with poorer health and mortality outcomes [62, 63]. While some have suggested that existential loneliness is irresolvable [33], a portion of participants described ways to resolve the experience through positive thinking and shared experiences. In these cases, positive outcomes such as acceptance and self-knowledge were possible for some individuals, and these aligned with those suggested by existing literature and research with older adults [13, 21, 32]. However, this research indicated that existential loneliness was generally characterised by negative emotions. Consequently, it may be useful for research and interventions focusing on alleviating loneliness to consider existential loneliness as well as more socially driven dimensions.

### An existential dimension of the loneliness experience

This research provides support for a multidimensional conceptualisation of loneliness with existential loneliness as one dimension or type. While various research conceptualises loneliness as including social, emotional, and existential dimensions [10, 11, 13, 14, 17], this conceptualisation has received relatively little empirical attention and existential loneliness has been particularly neglected in loneliness research. This study suggests that loneliness dimensions are subjectively separable. Descriptions of existential loneliness deviated from conceptualisations of social and emotional loneliness as described by Weiss [8] and from some aspects of loneliness experiences described in qualitative synthesis [7]. Namely, existential loneliness was deeper, less context-bound, and involved concerns about meaning and purpose, as opposed to relating more specifically to deficiencies in intimate relationships (emotional loneliness) or the larger social network (social loneliness).

Participants described existential loneliness as deeper, longer-lasting, harder to resolve, and less context-bound than other loneliness experiences. However, it did appear that existential loneliness is a recognisable dimension of loneliness, including key aspects of loneliness that have been outlined previously: it is generally aversive, labelled as loneliness by individuals experiencing it, can be impacted by social relationships, is associated with poor mental health, and is one of multiple forms of loneliness [64]. Moreover, whilst the majority of individuals who reported existential loneliness had also experienced another form of loneliness, 7% of participants reported that they had only experienced existential loneliness, indicating that dimensions of loneliness are subjectively separable for individuals experiencing them. These findings provide empirical support for a multidimensional conceptualisation of loneliness including existential loneliness. They also indicate that this dimension of loneliness is more persistent, suggesting that interventions and policy aimed at loneliness may benefit from targeting existential loneliness specifically.

### The relevance of social relationships to existential loneliness

Relatedly, while definitions stressed that existential loneliness could occur despite the presence of others, it was clear that social relationships played an important role. Existential loneliness was described as less context-bound, persisting or arising without an obvious cause. However, in describing their experiences, participants often described times of objective isolation and aloneness. These elements indicate that existential loneliness is perceived as less tied to objective social network characteristics but is nonetheless impacted by social activity. However, it is not inherently linked to social isolation, as participants also described existential loneliness after periods of socialising. This was perhaps linked to the sense that others could not understand them and that they were separate despite the presence of objective social contact. Quantitative research suggests that having a smaller social network and not living with a partner each predict existential loneliness, although it reports inconsistent findings around having daily contact with one’s social network [6, 18]. Similarly, existential loneliness has been described as more internal, rather than specifically related to relationship quality [65]. While the subjective experience of loneliness is separable from the objective circumstance of social isolation, they are associated [4]. Existential loneliness may represent a form of loneliness which is less directly tied to objective characteristics of the network and impacted more by internal cognitive processes, given that it relates to other people and the world generally, as opposed to deficits in specific relationships. Indeed, existing research indicates that experiences of loneliness differ in the degree to which they are related to specific social relationships versus being a generalised experience [7, 8, 66]. Future research should assess the degree to which existential loneliness is impacted by objective network characteristics, relative to other forms of loneliness.

### Precipitating factors and duration of existential loneliness

Existential loneliness was experienced as constant by some participants, but many described it as a regularly occurring experience, highlighting its transience for some individuals. Existing loneliness research emphasises the role of prolonged loneliness on outcomes like mortality and mental health [62, 63]. For some people, it appears that existential loneliness may function as a trait or mood which is relatively consistent, whereas for others it may be more of a state or emotion experience which is precipitated by certain events. Indeed, it has been suggested that a trait disposition towards existential isolation may occur if existential isolation states occur often or cannot be relieved [67]. In the current study, existential loneliness was depicted as less context-bound, with a lack of a clear cause in many situations, but various precipitating factors were mentioned. Being isolated or alone, socialising, periods of stress, and poor mental health were identified as particularly relevant. Existential loneliness has been identified as relevant for individuals with mental ill health [68] and this research indicates that it could be precipitated by anxiety, depression, trauma, and periods of stress; further exploring how existential loneliness might act a cause, consequence, or factor in experiences of poor mental health may help to delineate the relationship between these constructs. Boundary situations, in which people experience urgent and significant life-changing events such as serious illness, suffering, and death, are theorised to bring about existential loneliness [33, 69] and this research suggests that challenging events, whether internal or external, may be particularly relevant for this dimension of loneliness.

Additionally, in a sample which included individuals from 16 to 72 years old, experiences of existential loneliness were described by a small proportion of individuals as occurring particularly during adolescence and young adulthood. Indeed, existential loneliness has been suggested to emerge in adolescence [44] and has been evidenced in adolescents and young adults [41, 45]. This may be related to the finding that existential loneliness could occur following strong emotions, given that adolescence is a period of increased emotional intensity and older adults are more proficient at emotion regulation [70, 71]. While there is little research focusing on existential loneliness in younger adults, this may be a pertinent developmental stage for these experiences which is deserving of further attention. As existential loneliness was described as long-lasting and potentially permanent in the current study, easing the experience in younger people may be particularly helpful to avoid ongoing distress and mitigate the personal and public health impact of loneliness.

### Limitations and future directions

Although the findings of this research extend our understanding of the lived experience of existential loneliness across the lifespan, there are a number of limitations. While the use of online methods represents a novel and effective way to collect qualitative data, it excludes individuals without internet access or proficiency, who tend to comprise groups impacted by other sources of social inequality [72] and may obscure some aspects of in-person data collection such as tone of voice. This may also have contributed to the smaller proportion of older adults in this sample, although this group are strongly represented in other research on existential loneliness [15, 32, 34, 36, 37, 39]. Future qualitative work might usefully focus on how mental health and trauma are involved in experiences of existential loneliness, while quantitative research is needed to clarify how well this conceptualisation of existential loneliness fits within a wider multidimensional model of loneliness and with dimensions such as social and emotional loneliness.

## Conclusions

In summary, our results suggest that existential loneliness is a deep and pervasive form of loneliness involving feelings of deep disconnection. Reflection, overthinking, and negative emotions appear to play a role in these experiences, as do periods of stress, trauma, and poor mental health. A portion of individuals who had experienced existential loneliness had never experienced a non-existential form of loneliness, indicating that this represents a subjectively separable loneliness experience. This aspect of loneliness is deserving of further attention, which was specifically indicated by comments from several participants. Future research, policy, and practice around loneliness should take into account this deeply impactful but often overlooked dimension to improve our understanding of loneliness and inform supports for people experiencing loneliness.

## Data Availability

The datasets generated and/or analysed during the current study are not publicly available as research participants did not consent to raw data transcripts being made available and the content may compromise participant confidentiality. Reasonable requests may be addressed to the corresponding author.
